# SOX2^+^ Cell Population from Normal Human Brain White Matter Is Able to Generate Mature Oligodendrocytes

**DOI:** 10.1371/journal.pone.0099253

**Published:** 2014-06-05

**Authors:** Jorge Oliver-De La Cruz, Josefa Carrión-Navarro, Noemí García-Romero, Antonio Gutiérrez-Martín, Elisa Lázaro-Ibáñez, Carmen Escobedo-Lucea, Rosario Perona, Cristobal Belda-Iniesta, Angel Ayuso-Sacido

**Affiliations:** 1 Centro Integral Oncológico Clara Campal (CIOCC) and Instituto de Medicina Molecular Aplicada (IMMA), Hospital de Madrid Foundation, Madrid, Spain; 2 Division of Biopharmaceuticals and Pharmacokinetics, University of Helsinki, Helsinki, Finland; 3 Nanomedicine Laboratory, Instituto Madrileño de Estudios Avanzados IMDEA nanoscience, Madrid, Spain; 4 Neurosurgery Department, Hospital Universitario la Fe de Valencia, Valencia, Spain; 5 Instituto de Investigaciones Biomédicas CSIC/UAM, Madrid, Spain; Massachusetts General Hospital/Harvard Medical School, United States of America

## Abstract

**Objectives:**

A number of neurodegenerative diseases progress with a loss of myelin, which makes them candidate diseases for the development of cell-replacement therapies based on mobilisation or isolation of the endogenous neural/glial progenitor cells, *in vitro* expansion, and further implantation. Cells expressing A2B5 or PDGFRA/CNP have been isolated within the pool of glial progenitor cells in the subcortical white matter of the normal adult human brain, all of which demonstrate glial progenitor features. However, the heterogeneity and differentiation potential of this pool of cells is not yet well established.

**Methods:**

We used diffusion tensor images, histopathology, and immunostaining analysis to demonstrate normal cytoarchitecture and the absence of abnormalities in human temporal lobe samples from patients with mesial temporal sclerosis. These samples were used to isolate and enrich glial progenitor cells *in vitro*, and later to detect such cells *in vivo*.

**Results:**

We have identified a subpopulation of SOX2^+^ cells, most of them co-localising with OLIG2, in the white matter of the normal adult human brain *in vivo.* These cells can be isolated and enriched *in vitro*, where they proliferate and generate immature (O4^+^) and mature (MBP^+^) oligodendrocytes and, to a lesser extent, astrocytes (GFAP^+^).

**Conclusion:**

Our results demonstrate the existence of a new glial progenitor cell subpopulation that expresses SOX2 in the white matter of the normal adult human brain. These cells might be of use for tissue regeneration procedures.

## Introduction

A significant number of neurodegenerative diseases that affect the central nervous system progress with a loss of myelin. Within this group of diseases, multiple sclerosis (MS) is the most common cause of neurological disability in young adults [1], [2] and is a candidate disease for the development of cell therapies. The mobilisation of endogenous neural/glial progenitor cells, the isolation of these cells, and their *in vitro* expansion and implantation in the same patient within the most invalidating of the chronic sclerotic MS plaques in the brain might be the most promising of approaches. This idea has been reinforced by a number of reports describing a pool of oligodendrocyte progenitor cells (OPCs) [3]–[5] within the parenchyma of the adult human brain, which might be responsible for the spontaneous myelination observed in patients with MS [6]. Therefore, the identification and isolation of the various subpopulations within the OPC pool and the evaluation of their potential for generating oligodendrocytes *in vitro* will be essential for modulating their migration, differentiation, and integration *in vivo* in the damaged brain area. Additionally, primary cell cultures represent an invaluable model for testing the responsiveness of OPCs to drugs and growth factors, as well as for accurately defining the differentiation processes that result in fully functional oligodendrocytes.

Various subpopulations of OPCs have been purified from adult human brain samples according to the expression of A2B5, CNPase, or PDGFRA [3]–[5], [7]–[9] and expanded *in vitro*. However, to our knowledge, the presence of a SOX2^+^ (SRY-like homeobox 2) glial progenitor subpopulation, which precedes the expression of these markers during the differentiation process to glial cells, has not been reported, although some preliminary studies claim the possibility of isolating SOX2^+^ cells from human white matter, among other brain regions [10]. This transcription factor plays critical roles throughout development and is required for the reprogramming of somatic cells in induced pluripotent cells (iPS) [11], [12]. SOX2 is necessary to maintain pluripotency in embryonic stem cells regulating key genes such as Oct4 and Nanog, and then to regulate sequential specification towards the epiblast and the neuroectoderm [13], [14]. Its expression becomes restricted to the proliferative foetal ventricular zone of the neural tube, and it is retained in adult neural stem cells [13], [15]–[19]. Similarly, SOX2^+^ cells have been described as neural stem cells in the human foetal ventricular zone [20], the adult human subventricular zone (SVZ) [21], and the subgranular layer (SGL) of the hippocampus [22]. Moreover, SOX2^+^ cells have been identified as Bergmann glia in the adult human cerebellum [23].

The aim of this study was to identify new subpopulations of cells within the OPC pool in the white matter of the adult human brain that had the potential to generate mature oligodendrocytes, either *in vitro* or *in vivo*. For the first time, we were able to isolate a cell subpopulation enriched in SOX2^+^ cells from adult human white matter, which expanded as floating sphere-like colonies. These cells acted as glial progenitors *in vitro*, generating immature (O4^+^) and mature (MBP^+^) oligodendrocytes and, to a lesser extent, GFAP^+^ astrocytes. Lastly, we found SOX2^+^ cells in the original tissue, most of them co-localised with OLIG2, but not with the primary microglial, neuronal, or oligodendroglial markers, and only a small fraction of the cells were GFAP^+^.

## Materials and Methods

### DTI and Ki67 Immunohistological Screening of Human Tissue

Twenty-four samples of adult human brain white matter of normal appearance were obtained from planned resections during anterior temporal lobectomy for the treatment of intractable epilepsy. The procedures were conducted at Hospital La Fe (Valencia, Spain) and were performed with informed patient consent that provided their written consent to participate and in accordance with the medical and science ethics board (Comité Ético de Investigacón Biomédica del hospital Universitario La FE (CEIB)) that specifically approved the present study.

In order to ensure the absence of abnormalities in the tissue samples used in the present study, we first analysed the samples with two different techniques: pre-surgical diffusion tensor imaging (DTI) and automatised Ki67 immunostaining.

#### Method of DTI analysis

DTI was used to infer ultrastructural changes in brain white matter tissue according to the diffusion of water molecules. The diffusion-weighted images from all the patients included in this study were acquired using a single-shot echo-planar imaging pulse sequence with the following parameters: TR/TE of 10.100/102, acquisition matrix of 128×128, FOV of 250 mm, in-plane resolution of 2×2 mm and contiguous 2-mm slice thickness, 70 contiguous axial slices, and a voxel volume of 1.95×1.95×2 mm. The images were acquired with diffusion weighting in 30 non-collinear directions, all with a b value (diffusion weighting factor) of 1000 s/mm^2^. In addition, an image with no diffusion weighting (b value of 0 s/mm^2^) was acquired as a reference.

The DTI images were analysed using DTV.II SR toolbox software (Image Computing and Analysis Laboratory [UTRAD/ICAL] Department of Radiology, The University of Tokyo Hospital, extension of Volume 1, package [http://www.volume-one.org/]) with the following parameters to determine the degree of sensitivity for generating DTI tracts: Fractional Anisotropy (FA)  = 0.05, steps  = 160; Apparent Diffusion Coefficient (ADC) (x1K): any; So <80; Angle: any.

#### Histopathological study

The Ki67 immunohistochemistry was performed on a benchmark ULTRA using clone 30-9 (Ventana Medical Systems, Tucson, AZ), and interpreted with an iScan Coreo and Virtuoso algorithm (Ventana Medical Systems). A histopathological analysis of the tissue samples was performed to validate their use in the rest of the study. The architectural disorganisation of the cortex and neuronal heterotopia were studied using NeuN immunostaining. Microglia activation was examined by Iba-1 marking. The experiments were performed using the protocols described later.

### Cell Isolation Protocol and Culture

All samples were processed within 12 hours of extraction following a previously described protocol [24]. Briefly, brain white matter was macroscopically separated from temporal grey matter (**Figure S1**), minced, and washed in HBSS (Hanks balanced salt solution) w/o Ca2^+^/Mg2^+^. Enzymatic digestion was sequentially performed with Solution I (Papain [14 U/mL, Sigma] and DNase I [10 U/mL, Sigma] in PIPES solution) for 90 minutes at 37°C and Solution II (Papain [7 U/mL] and DNase I [15 U/mL] in PIPES: proliferative media [1∶1]) for 30 minutes at 37°C. The cells were then dissociated using diameter-decreasing polished Pasteur pipettes and filtered through a 70-µm mesh. Foetal bovine serum (10%, Invitrogen) was added to stop enzymatic digestion.

In 10 of the processed samples, the tissue disaggregates were resuspended in 0.9 M sucrose in 0.5x HBSS w/o Ca2^+^/Mg2^+^ and centrifuged at 750 g for 10 minutes [25]. The dissociated cells were resuspended in defined proliferative media and were maintained using standard procedures. Four viable cell cultures were obtained without sucrose gradient separation and 10 cell lines from the procedure with sucrose gradient.

The dissociated cells were resuspended in defined proliferative media composed of DMEM-F12, non-essential amino acids (100 µm), HEPES (2 mM), D-glucose (30 mM), bovine serum albumin (0.001%), sodium pyruvate (1 mM), l-glutamine (2 mM), N2 supplement (1X), penicillin-streptomycin-fungizone (1X) (all from Gibco-Invitrogen); and hydrocortisone (300 ng/mL) and triiodothyronine (30 ng/mL) from Sigma. The medium was supplemented with basic fibroblast growth factor (bFGF) (10 ng/mL, Sigma) and epidermal growth factor (EGF) (10 ng/mL, Sigma), and was replaced every two days.

When the cells growing in the monolayer adherent culture reached a confluence near 90%, a subculture was performed using Triple Express (Invitrogen) for 5 minutes at 37°C. The cells were reseeded at a density of 2000 cells/cm^2^ in 25 cm^2^ culture flasks. Using this procedure, all the cell lines could be expanded for at least seven passages.

The sphere-like clusters obtained using the protocol with the sucrose centrifugation were passaged before becoming necrotic by mechanical disaggregation with polished Pasteur pipettes, and planted at 3000 cells/cm^2^ on 24-well plates. All the cell lines could give rise to tertiary neurospheres. Additionally, adherent culture colonies derived from neurosphere cultures were subcultured separately according to monolayer culture protocol, and were able to reach a minimum of five passages.

**Figure 1:**
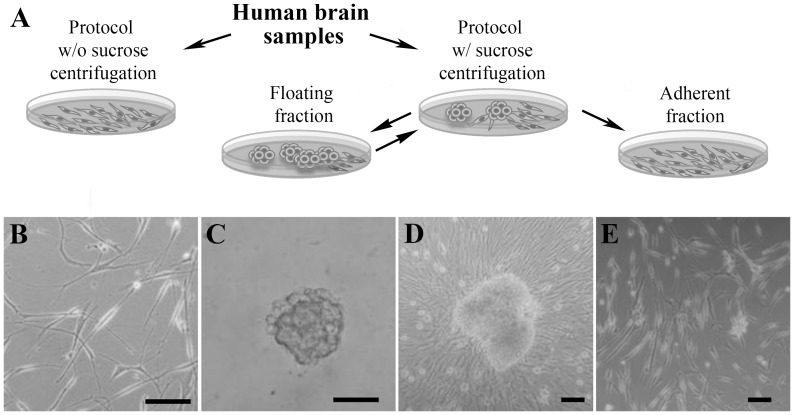
Proliferative cells can be isolated from adult human white matter. **A**. Diagram depicting the different procedures followed in the present work for the isolation of proliferative cells from human white matter. **B**. The cell isolation protocol without (w/o) the sucrose centrifugation step gives rise to a monolayer culture, which could be expanded more than seven passages. However, only 28.57% of samples generated viable cultures with this procedure (**B**). Therefore, we added a final sucrose centrifugation, which allowed the obtaining of sphere-like colonies lightly adhered to the culture plate from all samples processed (**D**). These cells generated an adherent culture that could be expanded independently for more than five passages (**E**). When the initial spheres were passaged and reseeded at a cell density of 3000 cells/cm^2^ or higher, new spheres were obtained (**C**), which, in turn, generated a new monolayer culture. These spheres could be passaged up to tertiary spheres, similarly to other proliferative cells isolated from adult human white matter. The scale bars represent 50 µm.

Three adherent culture cell lines produced by the spheres were seeded on ultra-low attachment at a density of 3000 cells/cm^2^, with or without 100-µM β-mercaptoethanol (Sigma) to test if they were able to regenerate floating cultures [26]. Viability was studied using Calcein/I Propidium iodide. After two weeks, the cells were seeded on Matrigel-coated coverslips, cultured for 24 hours, and fixed for SOX2 immunostaining (**Figure 1A** displays a schematic representation of the different cell cultures).

### Cell Differentiation

Spontaneous differentiation was assayed on small spheres formed two days after disaggregation, corresponding to four samples in triplicate. The spheres were seeded on matrigel-coated coverslips and maintained in growth media for 14 days prior to fixation.

A protocol directed to induce final oligodendroglial differentiation was tested in four samples in triplicate as previously described. The spheres were disaggregated into a single-cell suspension and seeded on a Matrigel-coated coverslip. The cells were maintained in an oligodendrocyte proliferative medium until confluence, composed of DMEM-F12, non-essential amino acids (100 µm), HEPES (2 mM), D-glucose (30 mM), bovine serum albumin (0.001%), sodium pyruvate (1 mM), l-glutamine (2 mM), N2 supplement (1X), and penicillin-streptomycin-fungizone (1X), all of which were acquired from Gibco-Invitrogen. The medium also included progesterone (300 ng/mL) and sodium selenite (30 ng/mL), which were acquired from Sigma. The medium was supplemented with bFGF (20 ng/mL, Sigma), platelet-derived growth factor alpha (PDGFa, 20 ng/mL, Sigma), and Neurotrophin-3 (2 ng/mL, Sigma). The medium was replaced every two days. When the cells reached confluence, the medium was supplemented with foetal bovine serum (2%, Gibco) and triiodothyronine (30 ng/mL, Sigma), and the cells were maintained one more week prior to fixation.

### Flow Cytometry

Twenty-four hours after cell isolation with the protocol, including the centrifugation in the sucrose solution, three cell samples were lightly disaggregated with polished Pasteur pipettes and washed once with 0.1 M PBS. The cell suspension was then incubated in A2B5 hybridoma supernatant (ATCC) for 30 minutes at 4°C, washed with 0.1 M PBS, 0.5% bovine serum albumin three times, and finally incubated in a secondary antibody (Alexa Fluor 488 goat anti-mouse, 1∶500 in 0.1 M PBS [Invitrogen]) and propidium iodide. After a final wash, the samples were analysed using a Cytomics FC 500 (Beckman Coulter) flow cytometer.

### RT-PCR and QRT-PCR

The total RNA was extracted from three cell lines of each culture type in passage 2 (spheres) or passage 3 (adherent cultures) using RNeasy, followed by DNase I treatment, both according to the manufacturer’s specifications (Qiagen). The cDNA was synthesised using a High Capacity cDNA Reverse Transcription Kit (Applied Biosystems). The molecular analyses were performed by PCR amplification, using primers designed with Primer 3 software (**Table S1**), followed by electrophoresis in 1.8% agarose gel. SYBR Green-based QRT-PCR (Applied Biosystems) was run in a LightCycler 480 Instrument (Roche). The amount of *SOX2* cDNA was normalised to the quantity of three housekeeping gene (β-actin, β-2-microglobulin, and GAPDH) transcripts.

### Immunocytochemistry

The cells were plated for 24 hours on Matrigel-treated coverslips, fixed with 4% paraformaldehyde in 0.1 M PB (Panreac) for 20 minutes, and washed with Dulbecco’s phosphate-buffered saline (DPBS, Invitrogen) w/o Ca2^+^/Mg2^+^.

The cells were blocked with 10% donkey serum (Jackson ImmunoResearch) and 0.1% Triton X-100 (Sigma) in 0.1 M DPBS for 45 minutes. Goat anti-human SOX2 antibody (1∶50, Chemicon), mouse anti-GFAP (1∶500, Dako), mouse anti-MAP2 (1∶200, Sigma), rat anti-MBP (1∶200, Sigma), and/or mouse anti-human Ki67 antibodies (Dako, 1∶250) were applied for 1 hour at 25°C in a blocking solution. For Ki67 immunostaining (rabbit anti-human Ki67, 1∶250, Dako), antigen retrieval was previously performed by exposing coverslips to boiling 10 mM sodium citrate, 0.05% Tween 20, pH 6.0 for 15 minutes.

A2B5 and O4 immunostaining was performed using live cells. The cells were washed, blocked with 5% goat serum in 0.1 M DPBS for 30 minutes at 4°C and incubated in primary antibody (A2B5 clone 105 hybridoma supernatant [ATCC, 1∶1]; anti O4 [Millipore, 1∶50]) for 45 minutes at 4°C. After washing, the cells were fixed with 4% PFA for 10 minutes and washed with 0.1 M DPBS.

Finally, for all cases, after washing, secondary antibodies were applied for 1 hour in DPBS (Texas Red donkey anti-goat antibody, 1∶150; DyLight 488 Donkey anti-mouse, 1∶400 [both from Jackson InmunoResearch]; Alexa Fluor 488 goat anti-mouse IgM, 1∶500; Alexa Fluor 555 donkey anti-goat, 1∶500; Alexa Fluor 488 goat anti-rat, 1∶500; Alexa Fluor 488 donkey anti-rabbit, 1∶500; Alexa Fluor 647 donkey anti-mouse, 1∶500 [all purchased from Invitrogen]). The cells were washed, counterstained with DAPI, and mounted with FluorSave (Molecular Probes, Invitrogen). The same procedure was performed for U373 cells and/or glioblastoma multiforme cells as a positive control.

The images were collected with a Leica TCS SP2 AOBS (Leica Microsystems) inverted laser scanning confocal microscope. All the confocal images were obtained under identical scan settings. Eight-bit, 1024 × 1024-pixel images were collected for each preparation. The best focus was based on the highest pixel intensity. Imaging conditions were identical for all the images. The images were identically processed using MetaMorph Software (Molecular Devices).

For the cell recount, at least 10 different images (including at least five spheres, depending on their size) from three separate experiments were counted.

### Immunohistochemistry for SOX2^+^ Cells

Sections from four temporal lobe samples were fixed with 4% PFA for 36 hours. After washing, the sections were cryoprotected in a 30% sucrose solution overnight and cryosectioned at 14 µm. The sections were washed with 0.1 M PBS, and antigen retrieval was performed by immersing the sample in 10 mM sodium citrate, 0.05% Tween 20, pH 6.0 at 95°C–100°C for 1 hour. After permeabilisation treatment with 0.2% Triton X-100 in PBS for 45 minutes, autofluorescence was partially blocked using Sudan black staining. The sections were incubated overnight at 4°C with goat anti-human SOX2 (1∶25 in PBS; Chemicon), alone or in combination with mouse anti-Ki67 (1∶250, Dako), mouse anti-GFAP (1∶200, Abcam), mouse anti-NeuN (1∶800), rabbit anti-Iba-1 (1∶200, Wako), or mouse anti-CNPase (1∶200, Sigma). The samples were then washed and labelled with appropriate secondary antibodies (Texas Red-conjugated donkey anti-goat antibody (1∶150) and DyLight-conjugated Donkey anti-mouse antibody (1∶400) [both from Jackson ImmunoResearch]; Alexa Fluor 488-conjugated donkey anti-rabbit antibody [1∶500, Molecular Probes]) for 1 hour at room temperature. After washing, the nuclei were counterstained with DAPI and sections were mounted with FluorSave (Molecular Probes, Invitrogen). Surgical samples from human glioblastomas, which express high levels of SOX2, were used as a positive control (**Figure S6**).

The images were collected with an Olympus FW-100 inverted laser scanning confocal microscope. All the confocal images were obtained under identical scan settings. Eight-bit, 1024 × 1024-pixel images were collected for each preparation. The best focus was based on the highest pixel intensity. Imaging conditions were identical for all the images. The images were equally processed using FluorView FV1000 (Olympus).

### Statistical Analysis

The data presented in this article represent mean ± standard deviation and were calculated using Statistical Package for the Social Sciences (SPSS, IBM). The FA and ADC means from the epileptic and contralateral side were compared using Student’s t-test for paired samples (α  = 0.01).

## Results

### Surgical Samples of Brain White Matter from the Anterior Temporal Lobe of Epileptic Patients do not Display DTI or Histopathological Abnormalities

The human temporal lobe samples were obtained from patients with mesial temporal sclerosis (age 17–55 [mean value: 38.53±10.98] years, 11 males and 13 females) undergoing surgical resection (**Figure S1)**. Anterior regions of the temporal lobe were chosen according to their remoteness to the epileptic focus, the hippocampus.

**Figure 2:**
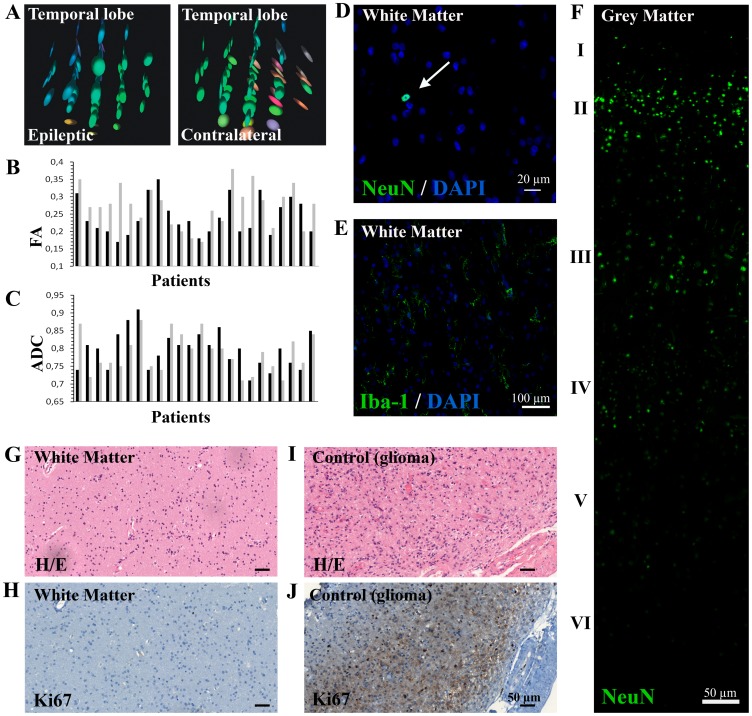
Brain white matter tissue obtained from epileptic patients does not show histopathological abnormalities. **A.** The DTI imaging analysis of the temporal areas used in our experiments showed diffusion tensors with elongated ellipsoidal form and grouped colouring, indicating a conserved white matter ultrastructure similar to the contralateral areas. **B–C.** The anisotropy of water molecules, quantified by the fractional anisotropy mean (FA) and the apparent diffusion coefficient mean (ADC), showed no significant differences between both epileptic (black) and contralateral (grey) white matter zones (p-value: 0.155 and 0.439, respectively) **D.** No neuronal heterotopia was observed within the white matter, with only a few neurons NeuN^+^ rarely spread throughout white matter parenchyma (white arrow). The scale bar represents 20 µm. **E.** Microglial Iba-1^+^ cells showed mainly quiescent morphology, with long branching processes and small cellular bodies. The scale bar represents 100 µm. **F.** Cortical disorganisation was also discarded by NeuN immunohistochemistry, as the neuronal layer could be perfectly differentiated (indicated by the roman numerals). The scale bar represents 20 µm. **G–J.** Haematoxylin-eosin staining revealed a normal cellularity (**G**) and the Ki67 recounts showed a normal number of cells in active phases of the cell cycle in all samples (**H**). Glioma tissue was used as positive control (**I–J**)**.** The scale bars represent 50 µm.

As a first approach, and to determine the presence or absence of abnormalities in the surgical brain tissue used for our study, the region of white matter from the anterior temporal lobe used in this study was compared with contralateral areas from the same patient using diffusion tensor images from the pre-surgical study. This process determined the integrity of the white matter architecture and connectivity according to the diffusivity of water molecules. The diffusion tensor matrix is visualised as an ellipsoid whose diameter in any direction estimates the diffusivity in that direction. As seen in the examples (**Figure 2A**), the prolate ellipsoids indicate that water diffusion is highly restricted towards their major principal axis, and, therefore, conserved white matter fibre tracts [27]. The preservation of axon organisation into tracts is indicated by the colour code, which represents the orientation of this primary axis.

Fractional anisotropy (FA) mean, a scalar value between 0 and 1 that measures the directionality of water molecule diffusion, showed similar values between the affected and the contralateral white matter (**Figure 2B**). Analogous results were obtained with the mean apparent diffusion coefficient (ADC), which takes into account that the diffusion process is not free in tissues (**Figure 2C**).

All the tissue samples displayed normal cytoarchitecture or mild gliosis and showed no evidence of the alterations that had been described in mesial sclerosis, including diffuse cortical architectural disorganisation (**Figure 2F**) of the cortex or neuronal heterotopia within the white matter (tested with the neuronal nuclei marker NeuN) (**Figure 2D**). No microglia activation was detected, as the cells displayed a resting morphology in the Iba-1 immunostaining (**Figure 2E**). Additionally, cell density showed normal values (**Figures 2G and 2I** **)**, and Ki67 recounts revealed a number of cells in the active cycle considered normal, thereby ruling out the presence of neoplastic proliferation (**Figures 2H and 2J**).

### Isolation of Sphere-like Colonies versus Monolayer Cells

The experiments were initially performed according to the stem/progenitor cell purification protocol used extensively for both adult human brain white matter and adult human neurogenic niches within the SVZ and SGL [9], [24], [28].

Using this procedure, only four of the 14 samples resulted in viable cultures (a 28.57% success rate). The dissociated cells generated adherent colonies that could be expanded as a monolayer, for a minimum of seven passages (**Figure 1B**). These cells suffered a decreasing proliferation rate through subsequent passages until they became quiescent.

To improve the efficiency of the isolation procedure, we introduced a final step, consisting of a sucrose gradient that helped to remove myelin and cell debris generated during mechanical disaggregation [25]. As a result, viable cultures were obtained from all samples processed (10 out of 10). Nevertheless, this modification led to the purification of a different cell type with a smaller size, which expanded, forming sphere-like colonies (**Figure 1D** **)**. Consistent with previous reports claiming limited self-renewal of adult glial progenitor cells [5], [9], the spheres could be passaged using mechanical disaggregation for at least two subcultures (tertiary spheres, but both their number and size decreased through successive passages) (**Figure 1C** **)**. Moreover, a seeding concentration of at least 3000 cells/cm^2^ was necessary to obtain viable cultures.

These neurosphere-like clusters were also able to generate a monolayer culture in each passage that could be propagated separately for at least five passages (**Figure 1E**).

### SOX2 Expression is Restricted to Sphere-forming Cells

To determine the phenotype of the isolated cells and the impact of the sucrose gradient in the cell type obtained, three cell lines from each type of culture were used to perform a molecular characterisation by PCR, using a set of primers designed to detect molecular markers that have been related to the neural stem cell phenotype in the bibliography (**Table S1**).

**Figure 3:**
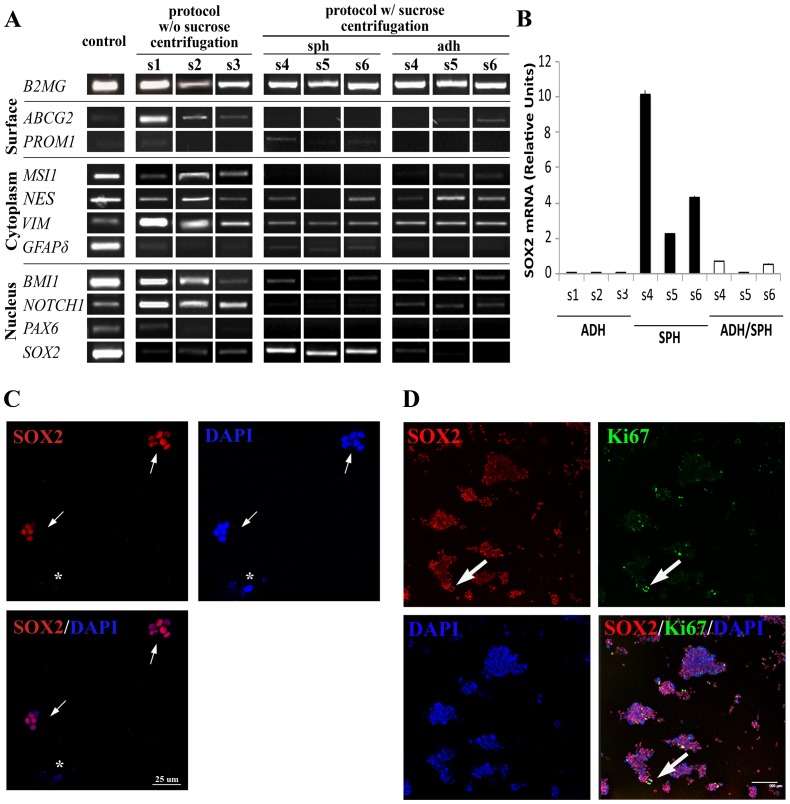
Cells isolated from adult human white matter differentially express neural stem cell related markers. **A.** The molecular characterisation by PCR of stem cell markers revealed a different pattern of gene expression among the three types of cell lines. Both adherent cultures types showed a very similar profile, and the sphere-forming cells differed in the absence of *ABCG2*, *MSI1*, and *PAX6*; the presence of *PROM1* in all samples; and, above all, a higher expression of *SOX2*. Primers, band size, and positive controls can be consulted in **Table S1. B.** QRT-PCR confirmed that spheres (SPH) express more *SOX2* mRNA than both adherent culture types. Bars represent mean ± standard deviation. **C.** After mechanical disaggregation to single cell suspension of primary spheres, small spheres started to grow, almost all the cells of which were SOX2^+^. The small white arrows indicate SOX2^+^ spheres, whereas the asterisk is marking a SOX2^−^ cell, which does not generate spheres. **D.** When these spheres became larger, the number of SOX2^+^ cells was reduced to 80.57% ±17.20%. All Ki67^+^ cells were SOX2^+^ (large white arrows). The scale bar represents 100 µm. The SOX2 protein could only be detected in the spheres (see **Figure S2)**.

The analysis revealed that all cell types express NSC cell markers (**Figure 3A**). In general, the three cell types coincide in the expression of the cytoskeleton markers vimentin (*VIM*) [29] and nestin (*NES*) [30], the transcription factor polycomb ring finger oncogene (*BMI-1*) [31], and *NOTCH1* [32], the transmembrane protein that can also act as nuclear factor. The isoform delta of glial fibrillar acid protein (*GFAPδ*) [33] could be detected in three samples, although it was more evident in the sphere lines. Nevertheless, different expression patterns for stem cell genes were confirmed among the three cell types. While both adherent cultures showed a very similar profile, the sphere-forming cells differed in the absence of ATP-binding cassette sub-family G member 2 (*ABCG2*) [34], musashi homolog 1 (*MSI*1) [35], [36], and paired box 6 (*PAX6*) [37]; the presence of CD133/prominin-1 (*PROM1*) [38]; and, above all, the more significant expression of *SOX2*. This enrichment in *SOX2* expression was also detected by QRT-PCR **(** **Figure 3B** **)**. Moreover, immunocytochemistry performed in all samples revealed that the SOX2 protein could be detected exclusively in spheres cultures, and not in the rest of the adherent cells **(Figure S2)**. Seven days after isolation, 43.69% ±8.92% of the cells were SOX2^+^. After sphere passage by mechanical disaggregation, small spheres start to arise, all containing almost exclusively SOX2^+^ cells **(** **Figure 3C** **and Figure S3)**. This number is reduced to 80.57% ±17.20% SOX2^+^ when the sphere size increases, although all Ki67^+^ proliferative cells were SOX2^+^ (17.45% ±5.80%) **(** **Figure 3D** **)**.

To determine whether the adherent cells that originated from spheres were able to recover the sphere phenotype, the cells were seeded in low adherence conditions [26]. Although a number of the cells survived and formed small cell aggregates under both conditions, SOX2 expression was not recovered, and the cells degenerated and died after two weeks (**Figure S4**).

### The Population Containing SOX2^+^ Cells Shows Glial Progenitor Cell Features

**Figure 4:**
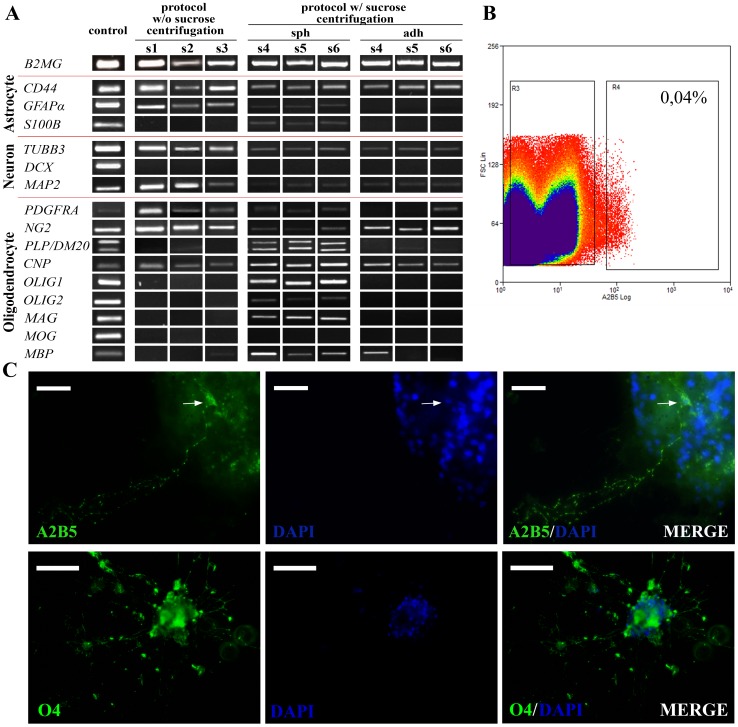
SOX2^+^-enriched cell population expresses glial progenitor signatures. **A.** The molecular characterisation by PCR showed that sphere-forming cells express a higher number of oligodendrocyte markers than either adherent culture (the cell monolayers obtained without the sucrose centrifugation step and those derived from spheres). In addition to the initial oligodendrocyte markers *PDGFRA*, *NG2*, and *CNP*, they express oligodendrocyte progenitor markers *OLIG1* and *OLIG2*, or the isoform *DM20* of *PLP*, and mature myelin proteins such as *PLP*, *MAG*, and *MBP*. Moreover, the three astrocytic markers (*CD44, GFAP*, and *S100B*) tested were also detected in the sphere culture. No difference in the neuronal markers was observed among the three culture types. **B.** FACS showed that only 0.04% of the isolated cells correspond to A2B5^+^/PI^+^ (region R4). **C.** Similarly, only a few A2B5^+^ cells were detected in primary spheres (the A2B5^+^ cell perinucleus in the image is indicated by the arrow), whereas more O4^+^ pre-oligodendrocytic cells were found. The scale bars represent 20 µm and 50 µm, respectively. Negative controls for FACS and immunocytochemistry can be found in **Figure S5**.

The molecular analysis demonstrated that SOX2 enrichment coincided with changes in the expression of glial genes (**Figure 4A**). In addition to the expression of early oligodendrocyte commitment markers, such as the alpha subunit of platelet derived growth factor receptor (*PDGFRα*), the proteoglycan *NG2*, and the enzyme 2′, 3′-cyclic nucleotide 3′-phosphodiesterase (*CNP*), the spheres expressed a higher number of oligodendrocyte progenitor markers, including the transcription factors *OLIG1* and *OLIG2*, which are necessary for oligodendrocyte differentiation, and the isoform *DM20* of the gene proteolipid protein (*PLP*) [39]. Moreover, the expression of the myelin-specific genes myelin acid glycoprotein (*MAG*), *PLP*, and myelin basic protein (*MBP*) was also detected, although not the gene myelin oligodendrocyte glycoprotein (*MOG*).

All the sphere samples expressed the three astroglial markers studied: the astrocyte precursor marker *CD44* [*40*], the calcium binding protein *S100B*, and the isoform alpha of the *GFAP* gene. Regarding the expression of neuronal precursor markers, microtubule associated protein 2 (*MAP2*) and β-3-tubulin (*TUBB3*) were detected in the three cell types, but not doublecortin (*DCX*).

The presence of A2B5 [41], the conventional marker to detect oligodendrocyte progenitor cells, and the pre-oligodendrocyte marker O4, was also confirmed by immunocytochemistry in initial sphere cultures, but it was undetectable in the other cell types (**Figure 4C** **and Figure S5B**). Nevertheless, the FACS analysis indicated that the vast majority of sphere cells isolated in the protocol with sucrose gradient were A2B5^−^ (**Figure 4B** **and Figure S5A)**. Thus, the cell population, which contained SOX2^+^ cells, seemed different from the A2B5^+^ population previously described, with about 100-fold fewer A2B5^+^ cells [9].

### SOX2^+^ Cells Generate Immature O4^+^ and Mature MBP^+^ Oligodendrocytes

**Figure 5:**
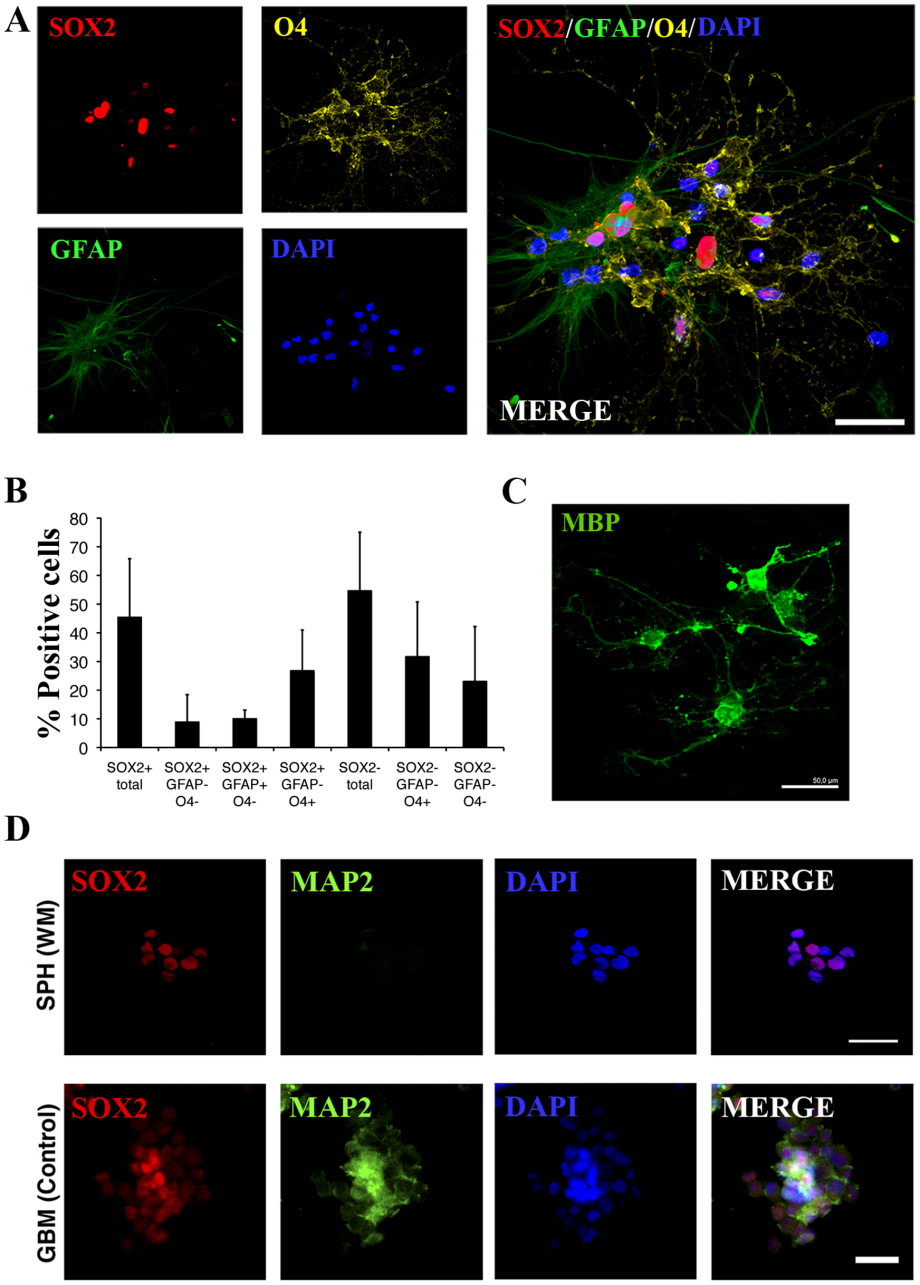
SOX2^+^ cells act as a glial progenitor *in vivo*. **A–B**. After the spontaneous differentiation protocol of small spheres, the number of SOX2^+^ cells decreased, accompanied by the emergence of O4^+^ cell processes, which correspond to 58.35% ±21.11% of total cells. A small number of GFAP^+^ cells could be also detected (10.01% ±3.06%), all of them SOX2^+^. The scale bar represents 25 µm. **C**. SOX2^+^ population exhibits morphology compatible with oligodendroglial differentiation after the directed differentiation protocol, which was confirmed by the detection of myelin protein MBP. The scale bars represent 50 µm. **D**. No neuronal MAP2^+^ cells were observed in the spheres after the spontaneous differentiation of sphere-forming cells. Primary cell cultures derived from glioblastoma were used as a positive control. The scale bars represent 20 µm.

To examine their spontaneous differentiation in media containing growth factors, small spheres were seeded onto Matrigel-coated coverslips and left to grow undisturbed for 14 days. During this time, cell extensions started to arise from the sphere, which slowed their growth, suggesting that the attachment was limiting cell proliferation. When immunostaining was performed, a decrease in the SOX2^+^ cell number was observed (45.38% ±20%). All the spheres were positive for the surface marker O4, one of the earliest antigens expressed in cells committed to the oligodendroglial lineage. Therefore, cell extensions corresponded to these small O4^+^ cells (58.35% ±21.11%), with 46.92% ±24.73% of them SOX2^+^/O4^+^ (26.72% ±14.25% of the total number of cells). We also found GFAP^+^ astrocytic cells (10.01% ±3.06%), all of which showed higher SOX2^+^ immunoreactivity (**Figures 5A–5B**). Note that, as shown in the image, the astrocytes were large, flattened, and expanded cells, with a larger nucleus, and tended to exit from the sphere core. Nonetheless, no MAP2^+^ neuronal/neuroblast cells were observed **(** **Figure 5D**). Finally, a directed differentiation procedure was performed, seeding single cells in oligodendrocyte proliferative medium until a high density was reached (which has been described as useful in achieving oligodendrocyte differentiation [9]), and the medium was then supplemented with foetal serum. All the surviving cells displayed an oligodendrocyte-like shape and positive immunoreaction directed against the myelin protein MBP, which could not be detected prior to the differentiation protocol (**Figure 5C**). Therefore, SOX2^+^ cells from adult white matter can be considered glial progenitor cells and are able to generate O4^+^ immature and MBP^+^ mature oligodendrocytes and, to a lesser extent, GFAP^+^ astrocytes.

### SOX2^+^ Cells are Present in Adult Human Brain White Matter

After the discovery of this SOX2^+^ cell subpopulation *in vitro*, we analysed four tissue samples of adult human white matter from the temporal lobe to determine whether these cells could be found *in vivo*. We detected the presence of SOX2^+^ cells in all the samples analysed. The cells rarely appeared, and were scattered throughout the white matter, although at times, the cells were concentrated in small areas. Their number was similar among the samples, representing approximately 2.01% ±0.73% of the total number of cells.

**Figure 6:**
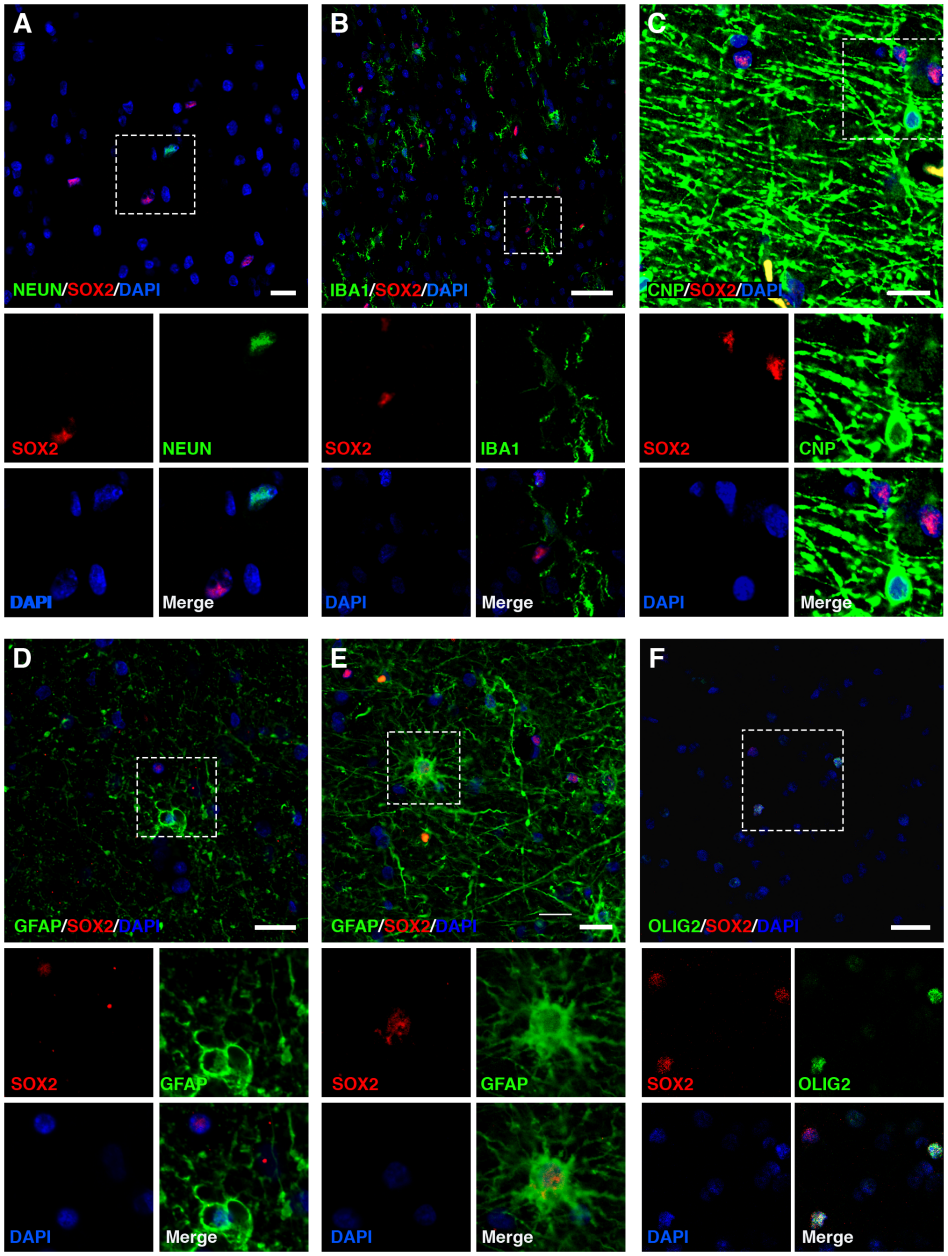
SOX2^+^ cells are present in adult temporal lobe white matter. SOX2^+^ cells can be found scattered within white matter. We do not detect NeuN^+^/SOX2^+^ neuronal cells (**A**), Iba-1^+^/SOX2^+^ microglial cells (**B**), or CNPase^+^/SOX2^+^ differentiated oligodendrocytes (**C**). Although the vast majority of SOX2^+^ cells were GFAP^−^ (**D**), some of them were GFAP^+^ astrocytes with more activated morphology (**E**). **F**. Finally, most of the SOX2^+^ cells corresponded to OLIG2^high^, which are thought to be oligodendrocyte progenitor cells in adult brain white matter. SOX2^+^/OLIG2^low^ and SOX2^+^/OLIG2^−^ could be also found in all samples. The scale bars represent 20 µm. Glioblastoma sections used as positive controls are collected in **Figure S6**.

Co-immunostaining with specific markers for the different cell lineages present in the brain was performed to determine the SOX2^+^ cell phenotype. No co-staining with Iba-1 was found, discarding the microglial identity of SOX2^+^ cells (**Figure 6A** **)**. Similarly, no co-localisation with CNPase or NeuN (**Figures 6B–6C**) was detected, which supports the idea that the SOX2^+^ cell subpopulation does not correspond to a differentiated oligodendroglial or neuronal population, respectively. On the other hand, the GFAP immunohistochemistry revealed a low proportion of co-immunolocalisation with SOX2^+^ cells in all the samples studied, always lower than the number of GFAP^−^ SOX2^+^ cells (**Figure 6D**). These GFAP^+^ SOX2^+^ (**Figure 6E**) astrocytes have many branching processes extended radially from the perinucleus, in contrast to the normal fibrous astrocytes present in white matter parenchyma.

Finally, as previously reported [42], [43], OLIG2 immunostaining distinguished two OLIG2^+^ cell populations (**Figure 6F**): the first, tenuously stained, corresponding to mature oligodendrocytes (OLIG2^low^); and the second, less abundant but expressing a higher level of OLIG2 (OLIG2^high^), identified as glial progenitors in adult human white matter. Interestingly, although we observed SOX2^+^ OLIG2^low^ and SOX2^+^ OLIG2^−^ cells, the confocal study revealed that most SOX2^+^ cells correspond to OLIG2^high^ cells.

Therefore, SOX2^+^ cells present in adult human brain white matter show features of glial progenitor cells that can be correlated with the SOX2^+^ enriched cell population isolated *in vitro*, which is able to generate O4^+^ immature and MBP^+^ mature oligodendrocytes and, to a lesser extent, GFAP^+^ astrocytes.

## Discussion

In this report, we describe for the first time the isolation of a SOX2^+^ glial progenitor cell subpopulation from adult human white matter. This small population of SOX2^+^ cells was also detected *in situ* and, in general, did not co-stain with any differentiated markers of the three main neuronal lineages or microglia.

SOX2 protein is involved in adult neural stem cell phenotype maintenance, proliferation, and subsequent differentiation [44]. In our *in vitro* studies, only the neurosphere-like clusters contained SOX2^+^ cells, a culture system commonly associated with neural stemness. These results agree with previous reports, in which SOX2 expression was shown necessary for the formation of spheres from adult human spinal cord tissue [45], human foetal VZ tissue [20], and rodent tissue [13], [16]. Furthermore, SOX2 induction is sufficient to transform human astrocytes into NSC-forming sphere-like colonies [46].

**Figure 7:**
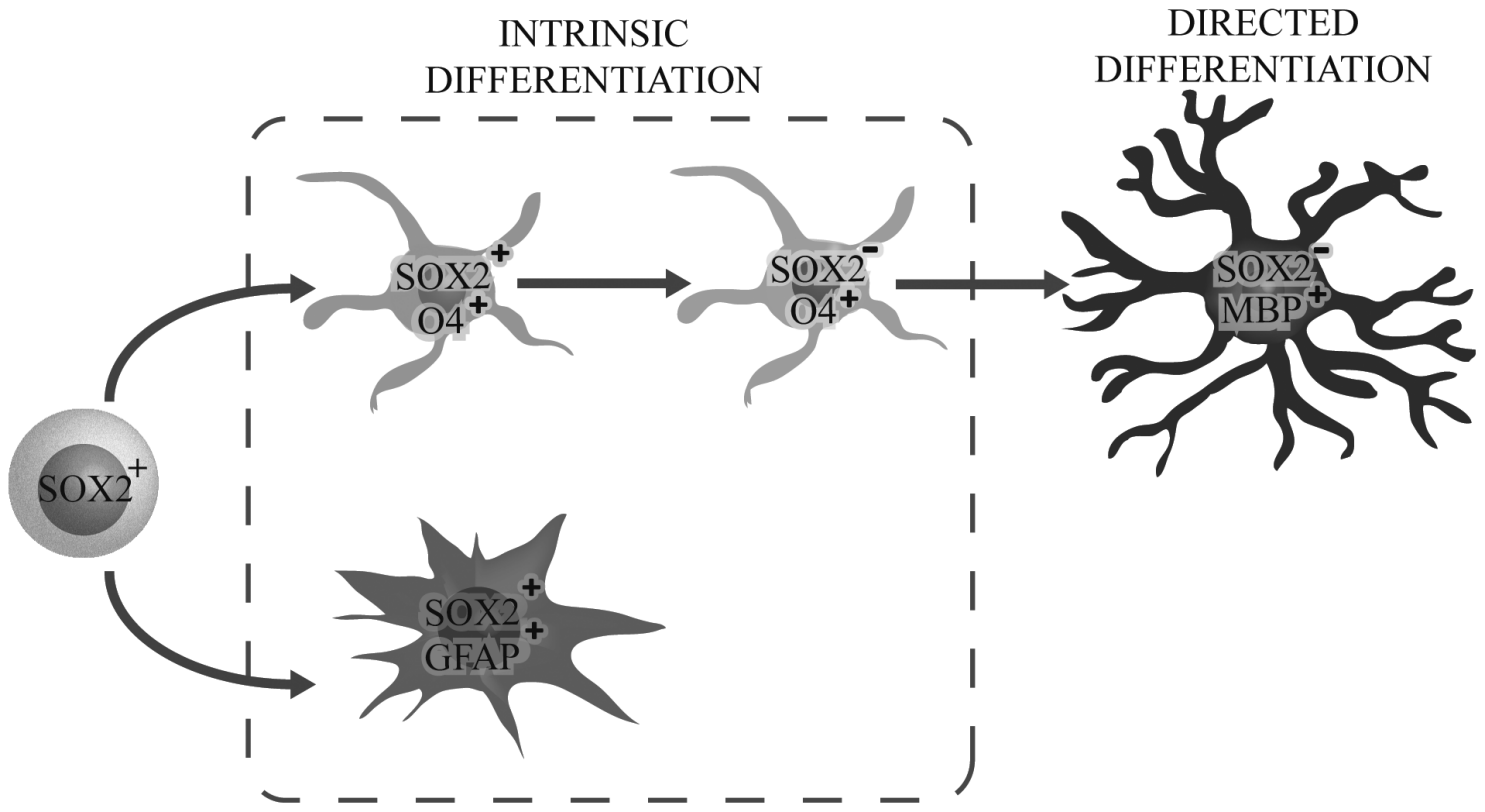
Schematic diagram representing our hypothesis about SOX2^+^ cell evolution *in vitro*. According to our findings, we hypothesise that SOX2^+^ cells correspond to glial restricted progenitors, spontaneously differentiating to O4^+^ pre-oligodendrocytes that finally lose SOX2 expression. In addition, a small number of GFAP^+^ astrocytes is generated. Finally, after exposure to the appropriate growth factors, SOX2^+^ cells are able to generate oligodendrocytes that express mature myelinating oligodendrocyte proteins such as MBP.

Nevertheless, sphere cultures are not exclusive to the neural stem cell phenotype, given that oligodendrocyte progenitors can also generate floating cell aggregates, known as oligospheres [47], [48]. Immunostaining revealed the presence of both glial populations, oligodendroglial and astroglial cells in the same sphere, but an absence of MAP2^+^ cells, indicating a bi-lineage competence, which is similar to that repeatedly established for previously described glial progenitors. This enrichment of glial gene expression in SOX2^+^ cells has also been found in foetal tissue [20] and adult SVZ tissue [21], where SOX2 expression has been associated with glial fate. It is known that SOX2 acts as a SOX11 activator, a transcription factor expressed in earlier oligodendrocyte progenitor commitment, and is lost after full differentiation. Other genes related to oligodendrocyte differentiation, such as PDGFRα, OLIG2, and NG2, have been found to respond directly to SOX2 variations [20]. According to our data, we hypothesise that SOX2^+^ cells correspond to the population of glial restricted progenitors which, in normal culture conditions, give rise to immature O4^+^ oligodendrocyte cells (that finally lose SOX2 expression), and a small percentage of astrocytic GFAP^+^ cells. Moreover, it is possible to induce their differentiation towards myelinating MBP^+^ oligodendrocytes with PDFGF/FGF/NT3 treatment followed by FBS exposure (**Figure 7**). However, we found an almost total absence of A2B5^+^ cells, whereas the glial progenitor populations already described were mostly overlapping and contained a big amount of A2B5^+^ cells [3]–[5].

This finding, together with the survival and proliferation of the isolated cell population in FGFb- but not PDGFa-supplemented medium, suggest that the SOX2^+^ population could correspond to a state prior to their commitment towards an oligodendrocyte progenitor cell (A2B5^+^/SOX2^−^) or even glial restricted progenitor cells (A2B5^+^/SOX2^+^) [49], [50]. Therefore, as SOX2^+^ cells have also been found to generate neurons under the appropriate conditions [10], [13], [20], we cannot reject the possibility that under specific culture conditions, our cells might behave as true neural stem cells *in vitro* and not just as glial progenitors. This same situation has been described for the A2B5^+^ OPC population [3]–[5].

SOX2 expression has also been related to a lower self-renewal *in vitro* in adult cells, a characteristic of progenitor cells. We were unable to expand SOX2^+^ cells for more than three passages, which, at first glance, might limit their use in cell replacement therapies. However, the presence of SOX2, together with increasing knowledge regarding the production of iPS cells using penetrating peptides (CPPs) [51], and the factors recently reported as modulating the biology of these SOX2^+^ cells [52]–[54], might help in the future to either modulate their behaviour *in vivo* or expand the SOX2^+^ progenitor cells *in vitro* before transplantation into chronic sclerotic MS plaques.

Beyond the discovery of these SOX2^+^ cells *in vitro*, we have also been able to identify SOX2^+^ cells in adult human normal-appearing brain white matter. Previous studies focusing on neoplastic or epileptic pathologies [55], [56] have shown that SOX2^+^ cells were present in the temporal cortex obtained from normal post-mortem tissue, but in what they considered to be negligible amounts. One of these analyses found a slight increase in SOX2^+^ cells in temporal mesial sclerosis, although it was not statistically significant in contrast to recounts performed in other epileptic pathologies.

During the characterisation of the SOX2^+^ cells, no co-immunolocalisation with neuronal or mature oligodendroglial markers was found, and only a small fraction of GFAP^+^ cells were discovered. This finding contrasts with the distribution reported in mice and rats [16], [17], in which virtually all SOX2^+^ cells scattered across the cortex corresponded to GFAP^+^ astrocytes. In our study tissue, SOX2^+^/GFAP^+^ might represent a reactive glial population due to epileptic injury, a factor that has been shown to be sufficient to reactivate SOX2 expression in quiescent astrocytes [57], [58]. This phenomenon might explain the slight increase in SOX2^+^ cells in mesial sclerosis. Thus, we may have a similar situation to that observed when oligodendrocyte progenitor cells were first described in human white matter, in which a small percentage of these cells were GFAP^+^ in epileptic samples; however, this phenotype was absent in normal tissue [3].

Nevertheless, the most relevant co-localisation of this study is the discovery of the OLIG2^high^/SOX2^+^ population. It has been reported that the OLIG2^high^ cell population–which expresses higher OLIG2 levels than mature oligodendrocytes–corresponds with practically all the proliferative cells present in adult human white matter [42], [43]. In mice, they generate oligodendrocytes as part of the normal replacement in the white matter. Furthermore, OLIG2^+^ cells are recruited by demyelinated plaques, even generating mature oligodendrocytes in early lesions [59]. Accordingly, this OLIG2^+^ SOX2^+^ cell pool could be responsible for the spontaneous remyelination observed in some patients [6], [60].

Therefore, according to their immunophenotype in the tissue, the behaviour of their *in vitro* counterparts, and the previous progenitor cells found in this tissue, our data suggest that this SOX2 population may represent a glial progenitor source within the adult human brain parenchyma. However, the limited availability of these precious adult human brain samples, both in size and number, reduces the number of assays and replicates that can be carried out, and further experiments to demonstrate the myelination capacity both *in vitro* and *in vivo* will be needed. The discovery of this new type of progenitor cell opens the possibility of designing cellular therapy strategies directed at mobilising these cells towards damaged areas to accomplish a functional cell replacement. Our isolated cells offer an *in vitro* model for further characterisation of their proliferative signal response and differentiation potential in order to modulate their activity *in vivo*.

## Supporting Information

Figure S1 **Sections of adult white matter from the temporal lobe obtained from patients who underwent surgical resection.** As observed in the examples, brain white matter can be visually distinguished from the cortical grey matter. This characteristic allowed not only the macrodissection performed in the cell isolation protocol, but also the identification of regions of interest in cryosections without the necessity of including specific markers.
(TIF)

Figure S2 **SOX2 is only detectable in sphere cultures.** Immunohistochemistry against SOX2 (red) and Ki67 (green) of adherent cells from the protocol without the sucrose gradient centrifugation (ADH, **A–B**) and adherent cells produced by sphere cultures (ADH SPH, E–H). No SOX2 was detected in either of the two samples. U373 cells were used as positive control (Control, **I–L**). The scale bar represents 20 µm.
(TIF)

Figure S3 **Small secondary spheres derived after mechanical disaggregation are mainly SOX2^+^.** The panel shows SOX2 immunostaining for 10 spheres from three different samples (1–3 correspond to the first sample, 4–6 to the second sample, and 7–10 to the third sample). Some cells show a high expression of SOX2 and others a lower expression. The scale bar represents 10 µm. The cell recount reveals that 97.14 were SOX2^+^ cells.
(TIF)

Figure S4 **Adherent cells generated from spheres cannot produce floating cultures. A.** Cells from adherent cultures were cultured in non-adherent conditions and maintained in media supplemented with growth factors for 14 days (n  = 3). During this time, some cell aggregates were observed, but they degenerated at the end of the experiment. Cell survival was studied using propidium iodide (**B**) and calcein (**C**). **D**. If these cell clusters were plated again in adherent conditions for 24 hours before fixation, they generated a monolayer, without the expression of SOX2 being detected by immunocytochemistry. The scale bar represents 100 µm (**E**).
(TIF)

Figure S5 **Negative controls for FACS analysis and A2B5/O4 immunostaining. A.** A small fraction of cells isolated using the protocol with the sucrose centrifugation was only stained with the secondary antibody, in order to delimitate the gates corresponding to real A2B5 staining. **B.** Similarly, when A2B5/O4 immunostaining was performed, a negative control without primary antibody was included to detect autofluorescence. As observed in the example, the primary spheres include an important quantity of cell debris in which fluorescence can be detected. The scale bar represents 50 µm.
(TIF)

Figure S6 **Glioblastoma multiforme tissue expressing high levels of SOX2 was used as a positive control.** Panel shows double immunostaining against SOX2 (red) combined with Iba-1 (**A–D**), CNPase (**E–H**), NeuN (**I–L**), and GFAP (**M–P**). The scale bars represent 100 µm (**D, H**) or 30 µm (**L, P**).
(TIF)

Table S1 **A set of primers for stem cell markers and markers for each neural lineage were designed using Primer 3 software.** After ensuring their efficiency, molecular analyses were performed by PCR amplification followed by electrophoresis in 1.8% agarose gel. Positive and negative controls were used for each marker according to the literature. *PLP/DM20 primers [61] generate two bands: one of them corresponding to the mature form *PLP* (247 pb) and the other to the isoform *DM20*, expressed in oligodendrocyte progenitor cells (120 pb).
(TIF)
