# Supplementary figures and images for: SOX2+ Cell Population from Normal Human Brain White Matter Is Able to Generate Mature Oligodendrocytes
Source: PLoS One. 2014 Jun 5;9(6):e99253. doi: 10.1371/journal.pone.0099253 (PMC4047120; doi:10.1371/journal.pone.0099253)

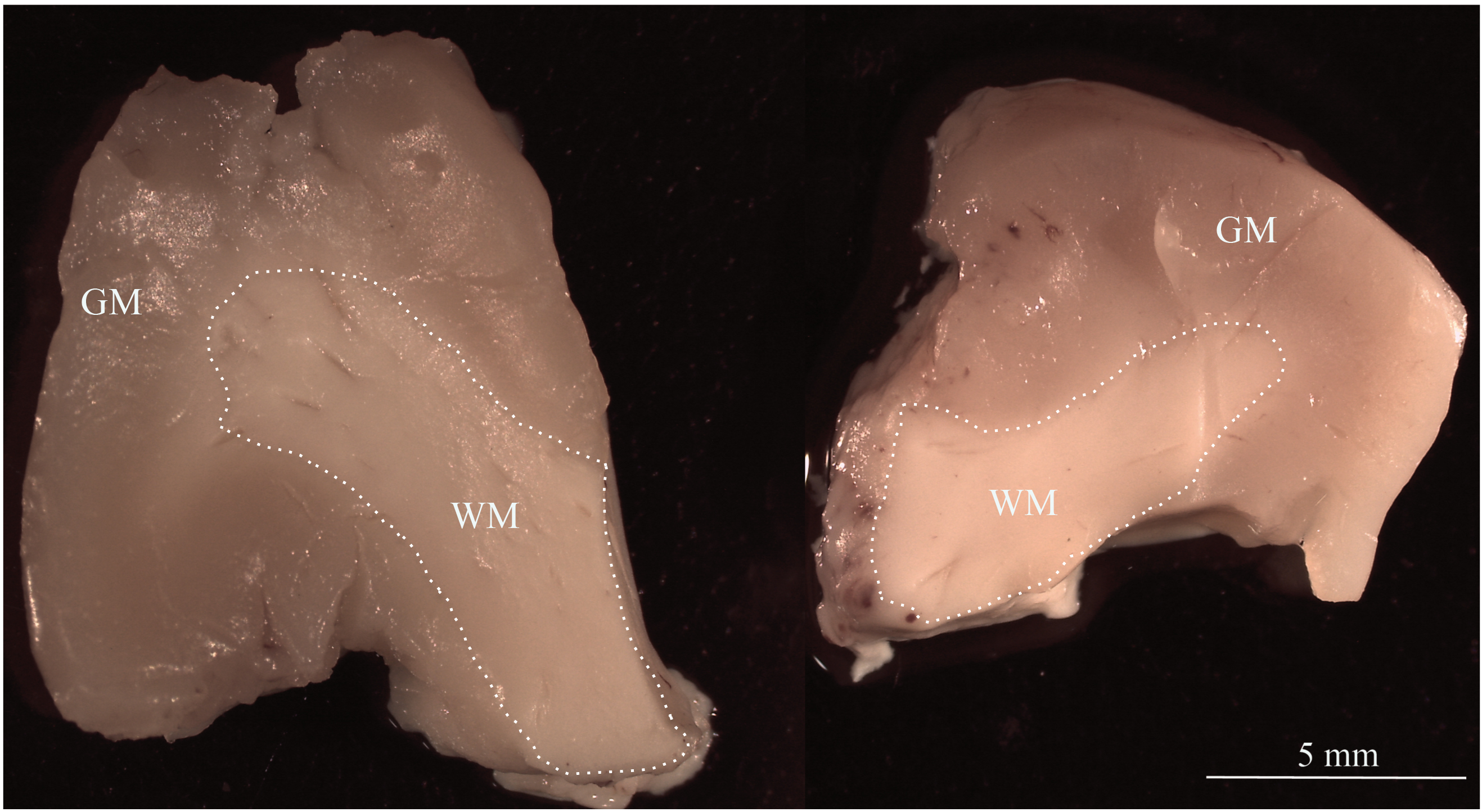

Supplement: Figure S1 — Sections of adult white matter from the temporal lobe obtained from patients who underwent surgical resection. As observed in the examples, brain white matter can be visually distinguished from the cortical grey matter. This characteristic allowed not only the macrodissection performed in the cell isolation protocol, but also the identification of regions of interest in cryosections without the necessity of including specific markers. (TIF) [file pone.0099253.s001.tif]

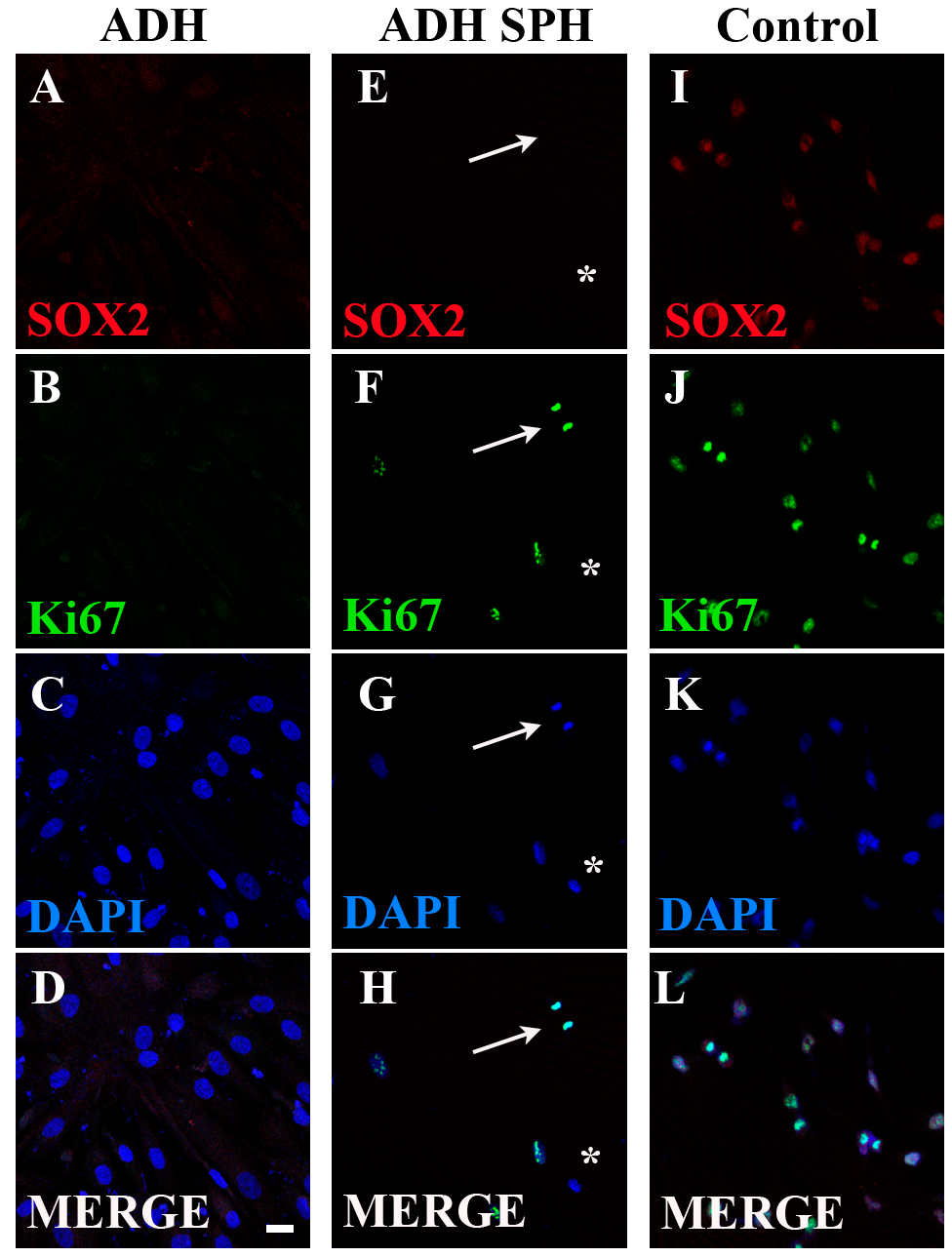

Supplement: Figure S2 — SOX2 is only detectable in sphere cultures. Immunohistochemistry against SOX2 (red) and Ki67 (green) of adherent cells from the protocol without the sucrose gradient centrifugation (ADH, A–B) and adherent cells produced by sphere cultures (ADH SPH, E–H). No SOX2 was detected in either of the two samples. U373 cells were used as positive control (Control, I–L). The scale bar represents 20 µm. (TIF) [file pone.0099253.s002.tif]

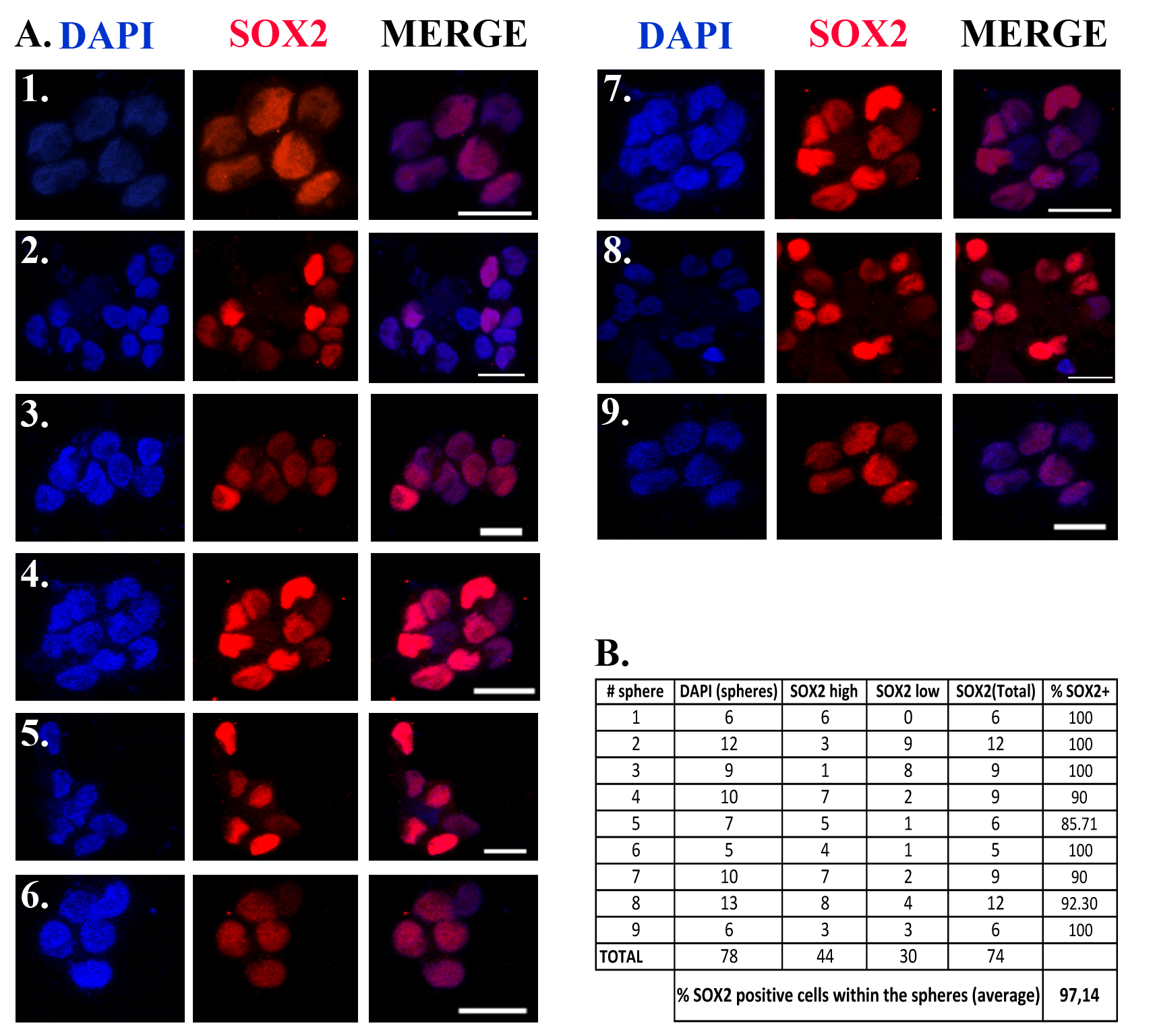

Supplement: Figure S3 — Small secondary spheres derived after mechanical disaggregation are mainly SOX2+. The panel shows SOX2 immunostaining for 10 spheres from three different samples (1–3 correspond to the first sample, 4–6 to the second sample, and 7–10 to the third sample). Some cells show a high expression of SOX2 and others a lower expression. The scale bar represents 10 µm. The cell recount reveals that 97.14 were SOX2+ cells. (TIF) [file pone.0099253.s003.tif]

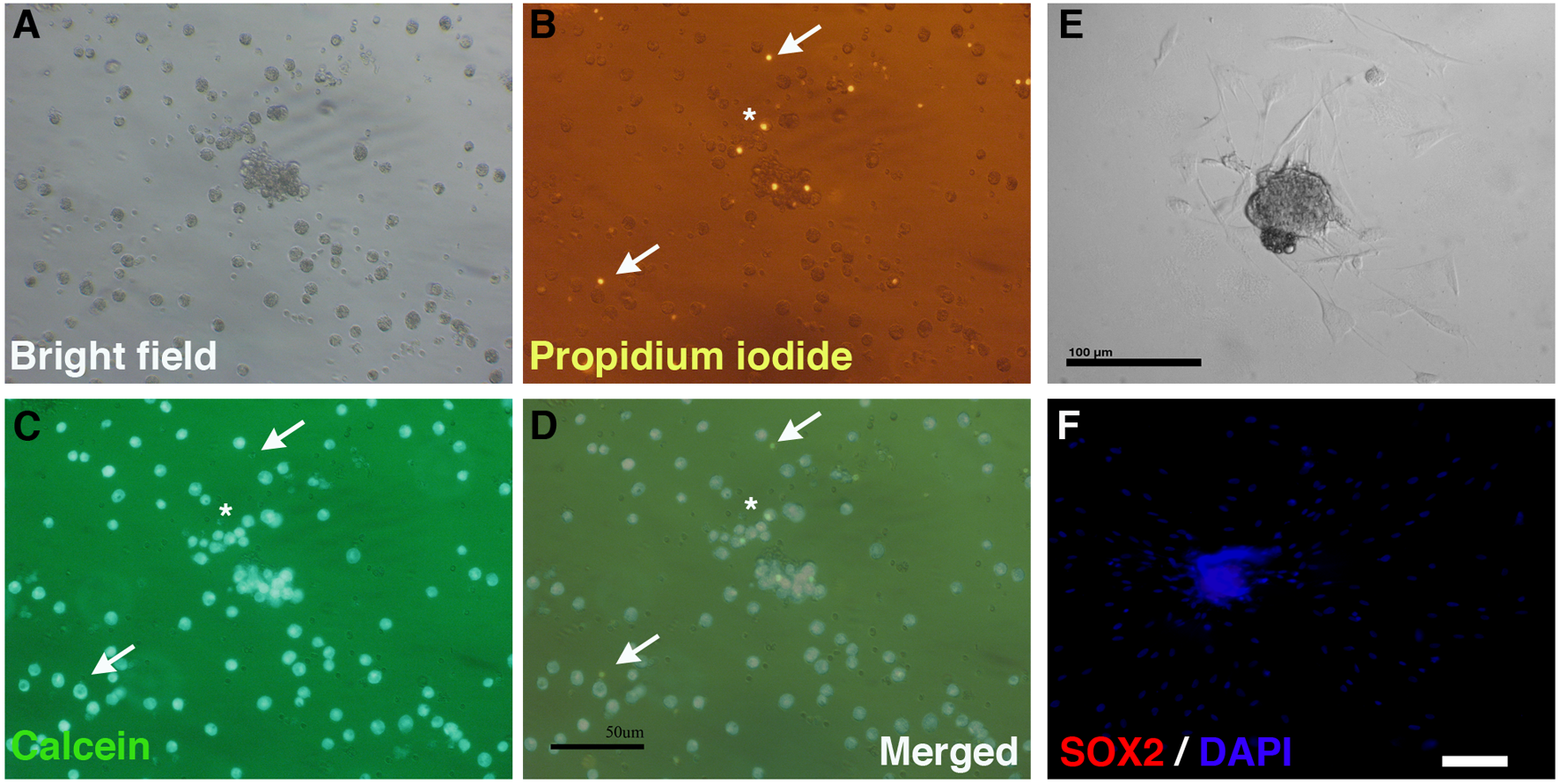

Supplement: Figure S4 — Adherent cells generated from spheres cannot produce floating cultures. A. Cells from adherent cultures were cultured in non-adherent conditions and maintained in media supplemented with growth factors for 14 days (n = 3). During this time, some cell aggregates were observed, but they degenerated at the end of the experiment. Cell survival was studied using propidium iodide (B) and calcein (C). D. If these cell clusters were plated again in adherent conditions for 24 hours before fixation, they generated a monolayer, without the expression of SOX2 being detected by immunocytochemistry. The scale bar represents 100 µm (E). (TIF) [file pone.0099253.s004.tif]

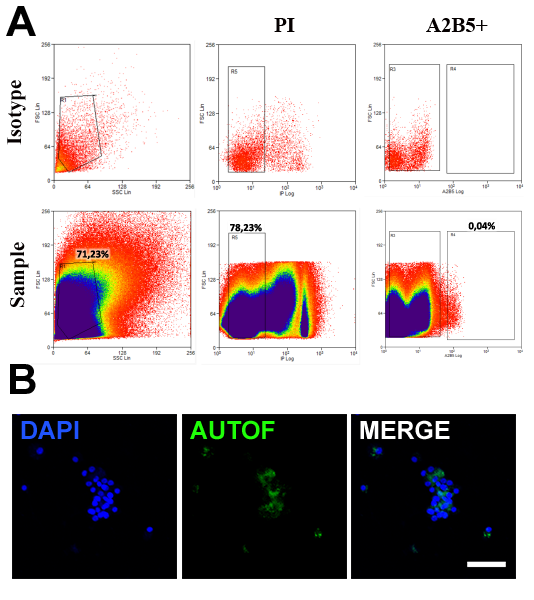

Supplement: Figure S5 — Negative controls for FACS analysis and A2B5/O4 immunostaining. A. A small fraction of cells isolated using the protocol with the sucrose centrifugation was only stained with the secondary antibody, in order to delimitate the gates corresponding to real A2B5 staining. B. Similarly, when A2B5/O4 immunostaining was performed, a negative control without primary antibody was included to detect autofluorescence. As observed in the example, the primary spheres include an important quantity of cell debris in which fluorescence can be detected. The scale bar represents 50 µm. (TIF) [file pone.0099253.s005.tif]

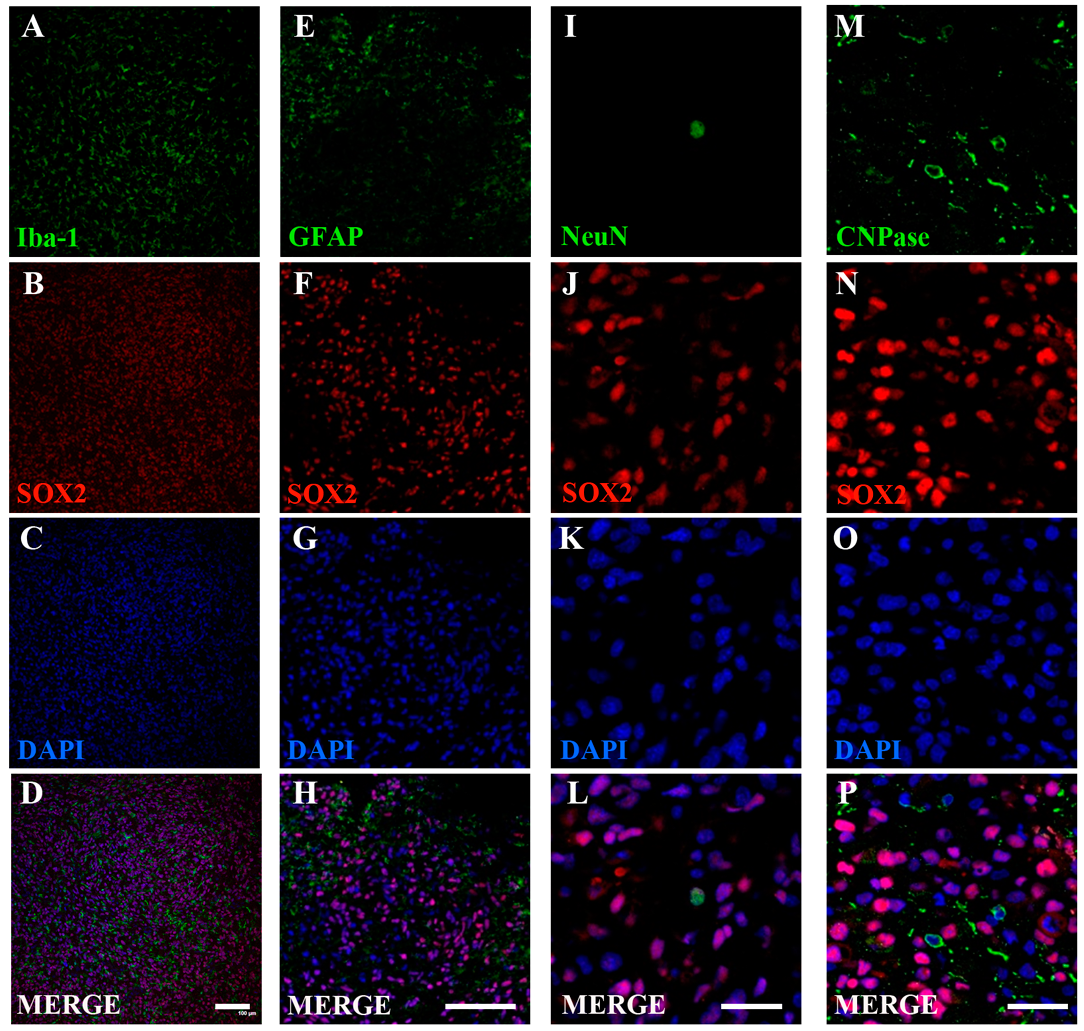

Supplement: Figure S6 — Glioblastoma multiforme tissue expressing high levels of SOX2 was used as a positive control. Panel shows double immunostaining against SOX2 (red) combined with Iba-1 (A–D), CNPase (E–H), NeuN (I–L), and GFAP (M–P). The scale bars represent 100 µm (D, H) or 30 µm (L, P). (TIF) [file pone.0099253.s006.tif]

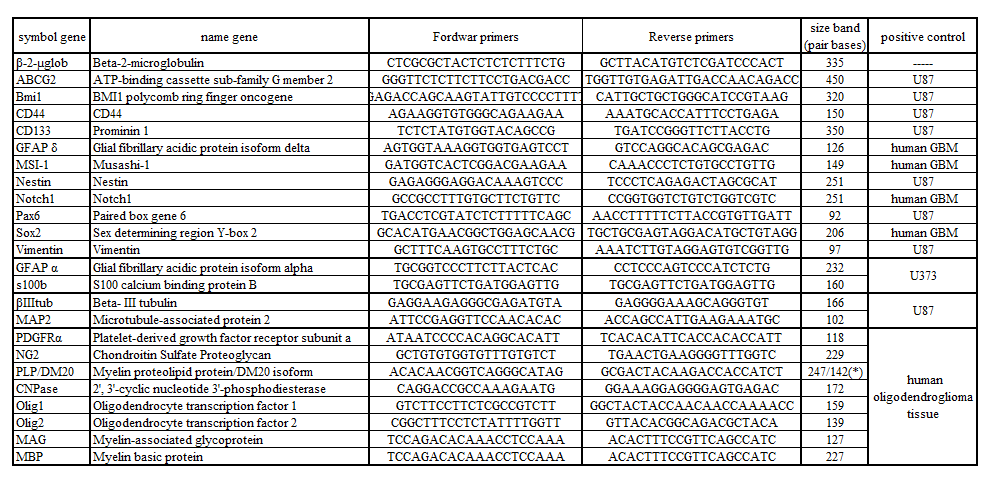

Supplement: Table S1 — A set of primers for stem cell markers and markers for each neural lineage were designed using Primer 3 software. After ensuring their efficiency, molecular analyses were performed by PCR amplification followed by electrophoresis in 1.8% agarose gel. Positive and negative controls were used for each marker according to the literature. *PLP/DM20 primers [61] generate two bands: one of them corresponding to the mature form PLP (247 pb) and the other to the isoform DM20, expressed in oligodendrocyte progenitor cells (120 pb). (TIF) [file pone.0099253.s007.tif]
